# Supplementary material for: Evidence supported by Mendelian randomization: impact on inflammatory factors in knee osteoarthritis
Source: Front Med (Lausanne). 2024 May 28;11:1382836. doi: 10.3389/fmed.2024.1382836 (PMC11165061; doi:10.3389/fmed.2024.1382836)
Supplement: Supplementary file 1 [file Data_Sheet_1.ZIP › data/GCST90274770_5e6/770.forest.pdf]

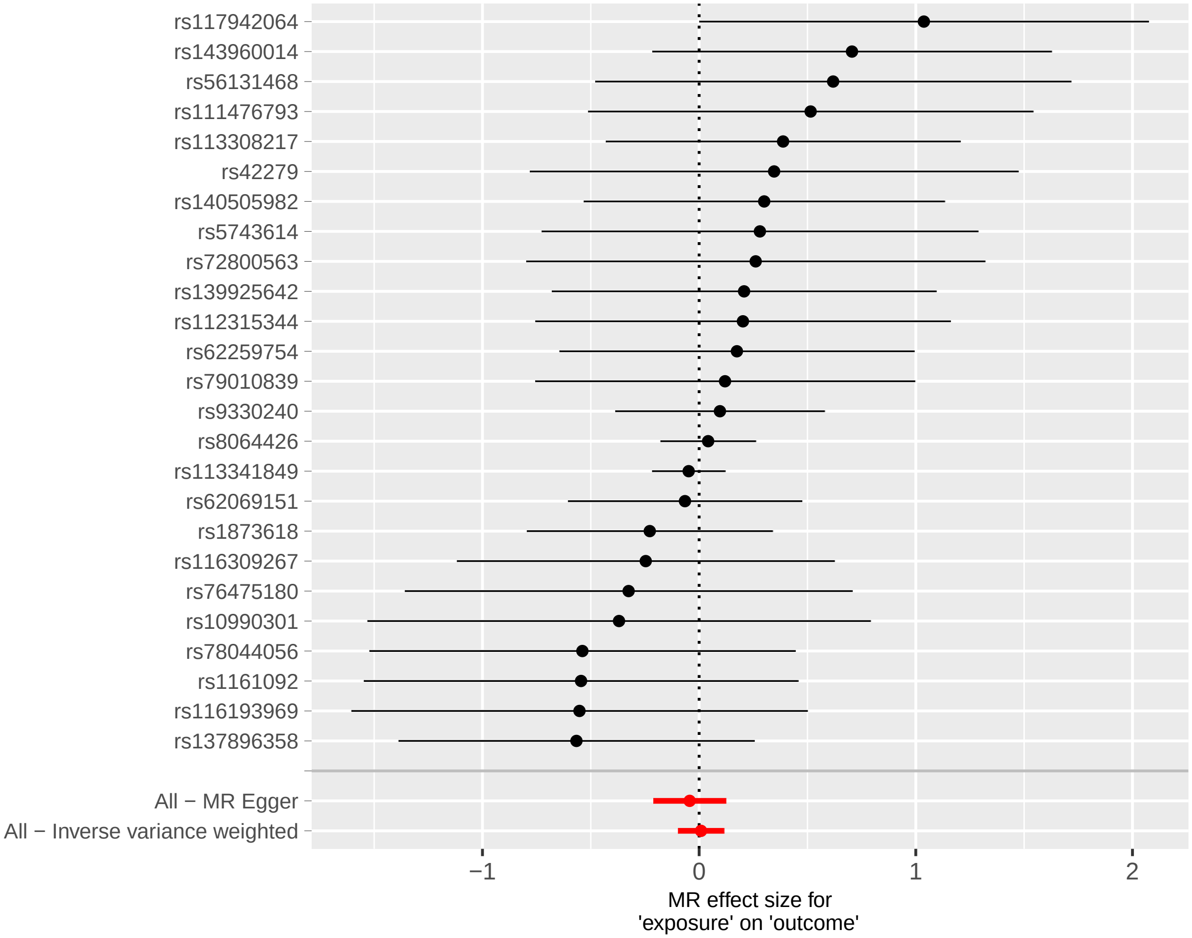

rs117942064
rs143960014
rs56131468
rs111476793
rs113308217
rs42279
rs140505982
rs5743614
rs72800563
rs139925642
rs112315344
rs62259754
rs79010839
rs9330240
rs8064426
rs113341849
rs62069151
rs1873618
rs116309267
rs76475180
rs10990301
rs78044056
rs1161092
rs116193969
rs137896358
All − MR Egger
All − Inverse variance weighted
−1
0
1
2
MR effect size for
'exposure' on 'outcome'
